# An exploration of attitudes regarding the use of a state tobacco Quitline for smoking cessation among low-income adults with a history of smoking

**DOI:** 10.18332/tpc/193572

**Published:** 2024-11-05

**Authors:** Alicia K. Matthews, Suchanart Inwanna, Jennifer Akufo, Cherdsak Duangchan, Safa Elkefi, Geri Donenberg

**Affiliations:** 1 School of Nursing, Columbia University, New York, United States; 2 College of Nursing, University of Illinois, Chicago, United States; 3 Ramathibodi School of Nursing, Faculty of Medicine Ramathibodi Hospital, Mahidol University, Bangkok, Thailand; 4 Chicago Department of Public Health, Bureau of Community Health - Office of Epidemiology, Chicago, United States; 5 Princess Agrarajakumari College of Nursing, Chulabhorn Royal Academy, Bangkok, Thailand; 6 College of Medicine, University of Illinois, Chicago, United States

**Keywords:** smoking cessation, state tobacco quitlines, federally qualified healthcare centers, health disparities

## Abstract

**INTRODUCTION:**

Smoking rates among low-income smokers are significantly elevated. State tobacco quitlines offer free and evidence-based treatments for smokers living in that state. This study investigated knowledge, attitudes, and beliefs associated with engagement with the Illinois Tobacco Quitline among confirmed smoking patients at a Federally Qualified Health Center (FQHC). Further goals were to obtain recommendations for strategies to improve patient awareness and engagement.

**METHODS:**

Data for this study were collected from August to October 2021 from patients receiving care in an FQHC in a large midwestern city in the USA. Clinic-based recruitment was used to enroll a sample of adult current smokers. In-depth interviews and brief surveys were completed with a volunteer sample of patients recruited from an FQHC. The interviews took approximately 60 minutes. Data analysis used descriptive statistics to summarize the responses to the study and deductive thematic analysis to analyze the qualitative interviews.

**RESULTS:**

Study participants (n=25) were primarily male, African American, and middle-aged (mean age: 52.5 years). The majority were daily smokers. Over half had heard about the Quitline from sources such as radio advertisements, but usage was low. Barriers to use included low motivation to quit, questions about effectiveness, and poor success with prior Quitline attempts. Participants described factors that would increase the appeal of the Quitline, including testimonials, personalization, and an empathetic approach. Participants were asked about the acceptability of receiving information about the Quitline via patient portals, and most were in support.

**CONCLUSIONS:**

Interventions are needed to raise awareness and utilization of Quitlines among patients receiving care in FQHC settings. Distribution of Quitline information via patient portals is an acceptable strategy for increasing awareness of services.

## INTRODUCTION

Adult cigarette smoking rates in the United States are at historic lows, with recent statistics suggesting that 11.5% of adults currently smoke^1^. The reduction in adult smoking rates stems from effective tobacco prevention and control activities such as health awareness campaigns, strict tobacco control regulations, and the widespread availability of smoking cessation aids^2^. Yet despite persistent tobacco control efforts, smoking and its related health disorders continue to pose significant challenges to low-income individuals^3^. For example, people impacted by poverty have higher tobacco use rates, fewer quit attempts, and are less likely to quit successfully, significantly increasing their risk of developing chronic health problems, including cardiovascular disease, chronic obstructive pulmonary disease (COPD), and various types of cancers^4^.

Poverty is also associated with factors that increase the risk of smoking initiation and maintenance^5,6^. For example, research indicates that smoking among low-income populations is related to low health literacy^6^. Furthermore, the prevalence of smoking is high among people living in poverty, and they are less likely to engage in evidence-based treatment and find quitting more challenging^7^. Beliefs about smoking and quitting are barriers to cessation^7^. In contrast, the affordability and availability of smoking cessation resources, such as counseling or medications, can be particularly challenging for low-income smokers, likely increasing the risk of continued smoking^7,8^. Critically, barriers to receiving appropriate care exacerbate smoking-related health inequities^9^. To address these disparities, it is essential to enhance the affordability, availability, and accessibility of smoking cessation treatments, and tailor interventions and strategies that overcome practical barriers faced by low-income smokers^10^.

Federally Qualified Health Centers (FQHCs) are crucial resources designed to provide healthcare services to low-income and uninsured patients, yet they remain underutilized for tobacco cessation efforts^10,11^. The median prevalence of tobacco use in FQHCs is 29.3%, compared to 20.8% in the general population^3,11,12^, underscoring the potentially high impact of FQHCs on underrepresented populations if smoking cessation services are adopted^11,12^. Unfortunately, few FQHCs implement evidence-based cessation programs, such as ‘Ask, Refer, and Advise’^13^, or deliver them consistently^14^ despite a mandate to do so. While ‘Ask’ is more commonly implemented, several factors contribute to the limited delivery of ‘Advise and Refer’ by FQHCs. For example, providers report insufficient time to deliver comprehensive smoking cessation counseling^15^, and healthcare providers’ attitudes of low priority towards smoking cessation can hamper patient efforts, in part due to provider unwillingness to address it during clinical encounters and provide counseling and referrals^16^. Further, if providers lack training and awareness of evidence-based smoking cessation techniques, they may promote ineffective strategies to quit and inconsistent referrals to or provision of smoking cessation treatment^16^.

Recognizing these challenges, there has been an increase in calls for collaboration between healthcare institutions and community resources such as Tobacco Quitlines^17^. Tobacco Quitlines are no-cost to the individual and offer proven and recommended therapies^13,18^, including ‘Ask, Refer, and Advise’. Although they provide individual-level treatment, the Quitlines are considered a population-based approach to increase tobacco cessation^19^ and are more accessible to individuals experiencing health disparities^20^. In addition, Quitlines offer free smoking cessation services in all fifty states. Free cessation assistance services are delivered through diverse channels, including nicotine replacement therapy, self-help materials mailed or delivered online, and on-demand treatment^13,21^. Perhaps most importantly, Quitlines can serve as an individual’s starting point for quitting smoking and accessing cessation medications^18^.

Smokers view Quitlines favorably, perceiving them as essential resources for quitting smoking and offering support throughout the process^21^. In 2019, an unheralded but remarkable milestone was achieved – the National Quitline Network (1–800-QUIT NOW) received its ten millionth call^22^. Despite their effectiveness and promise among smokers, Quitline reach and utilization remain low, particularly among low-income patients^21^. According to the yearly survey of Quitlines, only approximately 1% of US people who smoke used a Quitline in 2019, but when they did, 31.5% achieved at least 30-day abstinence at a 7-month follow-up assessment^23^. This study investigated knowledge, attitudes, and beliefs associated with engagement with the Illinois Tobacco Quitline among confirmed smoking patients at an FQHC. Further goals were to obtain recommendations for strategies to improve patient awareness and engagement.

## METHODS

### Study design

This mixed methods descriptive study was part of a larger project to increase access to smoking cessation treatments for low-income patients receiving care in a safety-net healthcare center or FQHC^24^. Qualitative methodologies are appropriate for obtaining patient-level data on healthcare service issues that are relevant and context-specific for them^25^. Data for this study were collected from August to October 2021. Informed consent was obtained from all participants. All methods for the study were approved by the Institutional Review Board at the University of Illinois Chicago (IRB #2020-1621).

Participants were current smokers who were patients at one of the participating healthcare systems clinics, aged ≥18 years and spoke English. A variety of outreach methods were used to recruit participants. First, potential participants were identified through the healthcare system’s electronic medical records. Then, two trained research assistants contacted eligible patients to explain the study and determine their interest in participation. Second, study flyers were placed in the clinical setting that described the study’s purpose, procedures, inclusion criteria, and contact information for the study coordinator. Third, patient navigators, nurses, and other healthcare providers distributed flyers to potential participants. Beginning in (August) 2021, we contacted 32 patients to invite their participation; 6 patients contacted our research team independently, and healthcare providers referred an additional 7 participants. A total of 25 patients who met inclusion criteria agreed to be interviewed and were scheduled.

### Data collection

Before the interview, participants completed online informed consent, provided permission to audio-record the interviews, and completed a brief (5–10 minutes) online survey via REDCap, a secure data collection platform. The brief survey included information about demographics, health, smoking habits and behaviors, and attitudes regarding the state quitline. Demographic questions in the survey addressed age, gender, race/ethnicity, sexual identity, education level, employment, health insurance, and health status. Smoking-related questions included types of tobacco products used, amount and frequency of use, number of peers who smoke, prior history of quit attempts and quit attempt methods, stage of readiness for smoking cessation, and use of the Illinois Tobacco Quitline (ITQL). We also asked about knowledge and the use of the MyChart patient portal to facilitate access to the ITQL.

We developed a semi-structured interview guide based on cognitive-behavioral models of health behavior change (i.e. Health Beliefs Models). The interview questions were designed to investigate knowledge, attitudes, perceived social norms, perceived risks and benefits, self-efficacy, barriers and facilitators related to smoking cessation and treatment, and engagement with the Illinois Tobacco Quitline.

Before data collection, two staff members conducted pilot interviews with research assistants to test the interview guide for length and clarity. The pilot test results were discussed with the research team, and the interview guide was improved. In-depth individual interviews were conducted via telephone (n=16) and Zoom (n=9) by two trained researchers and experience with qualitative research. The interviews were audio-recorded and lasted from 45 to 60 minutes. Each participant received $50 to complete the interview.

### Data analysis

Descriptive statistics (frequencies, percentages, means, and standard deviations) were used to summarize the survey data using SPSS statistical software version 26 (SPSS Inc., Chicago, USA). The interview recordings were transcribed verbatim by a professional service, and the transcripts were checked for accuracy by research team members. Interview data were then analyzed using deductive thematic analysis^26^. During this process, we read the transcripts several times to obtain an overall sense of the data and highlighted statements relevant to the research questions for coding. Second, codes with similar meanings were independently organized into subcategories. Third, the subcategories were compared and further sorted into appropriate and meaningful categories based on the fundamental concepts of this study. Finally, we compared the main categories from the analysis, discussing differences in the authors’ opinions until a consensus was reached. Throughout the data analysis, we worked collaboratively and iteratively to develop and revise the codes, subcategories, and categories.

### Ethical considerations

The study was approved by the University of Illinois Chicago Institutional Review Board (Protocol #2021-0578). To ensure participant confidentiality, a unique identification number was assigned to replace each participant’s name on all study materials.

## RESULTS

### Quantitative results

A total of 25 patients participated in the study. They ranged in age from 30 to 71 years (mean = 52.5, SD = 10.1), and 60% were male. Most participants were Black (76%), with the remainder being White (12%) and Hispanic (12%). Regarding education level, one participant had a Bachelor’s degree (4%), while the others had less than high school, and some college levels. All participants had health insurance. See Table 1 for a full description of participant demographics.

**Table 1:** Demographic characteristics of study participants (n=25)

*Characteristics*	*n (%)*
**Age** (years), mean (SD)	52.56 (10.15)
**Race/ethnicity**	
Black	19 (76.0)
White	3 (12.0)
Hispanic/Latino	3 (12.0)
**Gender**	
Male	15 (60.0)
Female	10 (40.0)
**Sexual identity**	
Lesbian	1 (4.0)
Gay	1 (4.0)
Straight	23 (92.0)
**Education level**	
Lower than high school	8 (32.0)
High school	8 (32.0)
Some college	8 (32.0)
Bachelor’s degree or higher	1 (4.0)
**Employment**	
Employed	9 (36.0)
Unemployed	15 (60.0)
Prefer not to answer	1 (4.0)
**Health insurance**	
Yes	25 (100)
No	0 (0)

Table 2 displays smoking behaviors and health conditions. Twenty-two participants reported smoking most days of the week (88%), averaging about 11 cigarettes daily. Sixty-eight percent of participants reported smoking their first cigarette within 30 minutes of waking up, indicating nicotine addiction. Most of the participants smoked mentholated cigarettes (84%). Twenty participants reported exclusively smoking cigarettes, while five reported dual use, including e-cigarettes, cigars, or cigarillos in addition to cigarettes. Most participants reported being in good to very good health (60%), but 52% had a health condition that was exacerbated by smoking.

**Table 2:** A summary of smoking behaviors and smoking health-related conditions of study participants

*Questions*	*n (%)*
**Have you smoked at least 100 cigarettes in your entire life?**	
Yes	25 (100)
No	0 (0)
**How many days of the week do you smoke cigarettes?**	
6–7	22 (88.0)
4–5	2 (8.0)
2–3	1 (4.0)
1	0 (0)
**On average, how many cigarettes do you smoke on the days you smoke?** mean (SD)	11.32 (8.06)
**How soon after waking up in the morning do you have your first cigarette?** (minutes)	
<5	7 (28.0)
5–30	10 (40.0)
31–60	4 (16.0)
>60	4 (16.0)
**What type of cigarettes do you usually smoke?**	
Menthol	21 (84.0)
Regular	2 (8.0)
Both	2 (8.0)
**How old were you when you first started smoking?** (years), mean (SD)	16.16 (3.27)
**Besides cigarettes, do you smoke any of the following?**	
None	20 (83.3)
E-cigarettes	2 (8.3)
Cigars	1 (4.2)
Cigarillos	1 (4.2)
**If you are in a relationship, does your partner smoke?**	
Yes	16 (64.0)
No	5 (20.0)
I am not in a relationship	4 (16.0)
**How many of your close friends smoke?**	
None	1 (4.0)
A few	12 (48.0)
Most	10 (40.0)
All	1 (8.0)
**Would you say that, in general, your health is?**	
Excellent	0 (0)
Very good	3 (12.0)
Good	12 (48.0)
Fair	8 (32.0)
Poor	2 (8.0)
**Have you ever been told by a healthcare provider that you have a smoking-related illness?** (For example, lung cancer or emphysema)	
Yes	3 (12.0)
No	22 (88.0)
**Do you have a health condition you have been told is worsened by smoking?** (For example, diabetes, HIV infection, high blood pressure, lung or respiratory illness, or heart disease)	
Yes	13 (52.0)
No	12 (48.0)

As shown in Table 3, 22 participants were advised by a health professional to quit smoking within the last 12 months. Of those, approximately 77.3% received information about stop-smoking counseling. Using various methods, 64% (n=16) attempted to quit smoking within the last 12 months. The most common method was ‘Cold Turkey’ at 56.3%, with 50% using nicotine patches or gums and 31.3% using a stop-smoking medication. Four participants felt they needed more time to quit smoking (16%), while sixteen felt prepared (64%). Most participants had heard of the Illinois Tobacco Quitline (64%); however, only 12.5% had used it.

**Table 3:** A description of participants’ smoking cessation behaviors, readiness to quit, and use of the Illinois Tobacco Quitline

*Questions*	*n (%)*
**In the past 12 months, did a doctor, nurse, or other healthcare team member tell you to stop smoking?**	
Yes	22 (88.0)
No	3 (12.0)
**If yes, did they give you information about stop-smoking counseling or medications?** (N=22)	
Yes	17 (77.3)
No	5 (22.7)
N/A	3 (12.0)
**In the past 12 months, have you tried to quit smoking?**	
Yes	16 (64.0)
No	8 (32.0)
No, I have not tried to quit, but I did cut back or try to cut back	1 (4.0)
**In the past 12 months, did you use any of the following to help you try to quit?** (Check all that apply) (N=16)	
Stop smoking class or support group	2 (12.5)
The Illinois Tobacco Quitline or similar telephone helpline	2 (12.5)
The nicotine patch or gum	8 (50.0)
A stop-smoking medication (Chantix, varenicline, Wellbutrin, or Zyban)	5 (31.3)
Self-help books or pamphlets	3 (18.8)
‘Cold Turkey’	9 (56.3)
A telephone or internet app	3 (18.8)
Other (praying, chewing tobacco, reading about the harmful effects of smoking as a person with diabetes)	3 (18.8)
None	1 (6.3)
N/A	9 (36.0)
**If you did not use any stop-smoking medications, why not?** (Check all that apply) (N=11)	
My doctor or nurse did not suggest I take them	7 (63.6)
Too many side effects	1 (9.1)
I am worried about becoming addicted to them	1 (9.1)
They are too expensive	1 (9.1)
They are not sold in stores near my home	2 (18.2)
Other (not ready to quit, already on several other medications; did not want to add yet another medication)	2 (18.2)
N/A	14 (56.0)
**I am ready to quit smoking**	
Completely disagree	1 (4.0)
Disagree	3 (12.0)
Neither agree nor disagree	5 (20.0)
Agree	10 (40.0)
Completely agree	6 (24.0)
**I feel confident I can quit smoking when I am ready to stop**	
Completely disagree	0 (0)
Disagree	9 (36.0)
Neither agree nor disagree	3 (12.0)
Agree	9 (36.0)
Completely agree	4 (16.0)
**I feel motivated to quit smoking**	
Completely disagree	0 (0)
Disagree	5 (20.0)
Neither agree nor disagree	5 (20.0)
Agree	12 (48.0)
Completely agree	3 (12.0)
**Have you ever heard of the Illinois Tobacco Quitline?**	
Yes	16 (64.0)
No	8 (32.0)
**Have you ever used the Illinois Tobacco Quitline?** (N=16)	
Yes	2 (12.5)
No	14 (87.5)
N/A	9 (36.0)

N/A: not applicable

### Qualitative findings

Table 4 provides a summary of the key qualitative findings related to the domains of interest, including: 1) awareness of the ITQL, 2) utilization of the ITQL, 3) perceptions about the ITQL among non-users, 4) desired characteristics of the Quitline experience, and 5) increasing awareness and utilization of the ITQL via the electronic health record. Main themes and subthemes are given below with illustrative quotations, as appropriate.

**Table 4:** A summary of the qualitative themes and subthemes of attitudes toward the use of the Illinois Tobacco Quitline for smoking cessation

*Main themes*	*Subthemes*	*Qualitative findings*	*Quotations*
**Awareness of the ITQL**	-	Several participants had heard about the ITQL, but only a few had direct experience using it.	‘I saw a commercial on TV.’ ‘[their providers] gave [them] pamphlets about it.’
**Utilization of the ITQL**	-	Readiness to quit and the experience of using ITQL did not work for them, obstructing their ability to utilize the Quitlines.	‘I guess because I know I have to and that I want to. I just haven’t taken that final step.’ ‘No, Because I was hearing that for a long time, … And it doesn’t work for me.’
**Perceptions about the ITQL among non-users**	-	The ITQL was seen as helpful for quitting; however, several participants were not ready to use it and were planning to utilize it when they were ready to quit.	‘Yeah, it sounds like something I would be interested in when I’m ready to quit.’ ‘If I was to get ready to stop smoking.’
**Desired characteristics of the Quitline experience**	Peer insights	Receiving advice from peers or former smokers as counselors would increase their trust, as they share the same smoking experience.	‘Well, if they hadn’t smoked, I wouldn’t be as receptive as I probably would have been …’
	Personalization	Personalizing the smoking cessation plan was thought to increase patient comfort.	‘How you talk to someone, they ask you about your tobacco use, then they set up a specific plan for you. I think that would draw a lot of people in.’
	Empathetic approach	Showing empathy toward smokers and a willingness to support them could greatly increase their trust in the initiative, while avoiding pressure or coercion when advising them to quit.	‘… knowing that you got somebody willing to help you, and concern, and show you – and give you help on trying to quit smoking. That’s not easy.’ ‘[providers should say] We’re not here to force you to stop smoking, but it’s good for your health, so we’re here to help you.’
	Outreach approaches	To effectively promote Quitline services, used multiple channels like advertisements, flyers, billboards, and social media platforms such as TikTok to reach a broader audience.	‘About now, I feel as though they put something on TikTok … If they happen to have something on there that catches a person’s eye, they would be catching a lot of people’s eyes.’
	Patient-centered communication	Healthcare professionals should use patient-centered communication, focusing on meaningful conversations and consistent, frequent contact to facilitate connection with the Quitline.	‘… I would say get more in depth with that conversation. Not to drag it on. But you know what I’m saying really [help connect people even to the Quitline person].’ ‘Constant communication, instead of the three months, I think it should be like once a month instead …’
**Increasing awareness and utilization of the ITQL via the electronic health record**	Collaboration, support and guidance	MyChart messages would help patients feel supported and advised while keeping control over their quitting process.	‘I think with the MyChart, … it makes me personally think like okay, the doctor looked at this, … and he thinks with the tobacco specialist, that this is best. So, that’s something that I would probably follow more.’
	Convenience and flexibility	Participants favored MyChart for its convenience, allowing easy access to resources when ready to quit or needing help, without much effort.	‘I think that would be a good idea because if they feel like they want the help, they can always go over to their MyChart and click on that button and say, hey, it’s that time. I think I want help.’
	Educating and monitoring quitting smoking	Using MyChart to promote smoking cessation could increase awareness and provide consistent reminders through notifications about the importance of quitting.	‘… doctors got something to pass on to the people that are not aware of it.’
	Facilitating access to care	Participants highlighted MyChart’s continuous and easily accessible services as another benefit.	‘If I called and then somebody didn’t answer, I could just go to MyChart and try that way.’

### Awareness of the ITQL

Based on survey responses, many participants had heard about the ITQL program, but few had direct experience using it. Participants reported learning about the ITQL from various sources during the qualitative interviews. The most common source was advertisements or commercials on TV or the radio, and less frequently, it was from their healthcare provider:

*‘I saw a commercial on TV.’*

*‘ … also commercials on the radio. Because I listen to the radio when I drive, you know.’*

Another source was healthcare providers and representatives as some doctors ‘gave [them] pamphlets about it’ and ‘some people came [there] where [they] lived and talked about it’. Furthermore, written public advertisements served as another source of information about the ITQL. One participant mentioned:

*‘I’ve seen signs, billboards, and stuff that [the ITQL] had.’*

### Utilization of the ITQL

Only a few participants reported contacting the ITQL for assistance with smoking cessation. For example, one participant used the ITQL by receiving nicotine patches and attending smoking cessation sessions:

*‘Mm-hmm … I think some people came here to where I live. And talked about it years ago. And, actually, I ordered some patches. They gave me some patches. And then we used to have people come to where I live and talk about smoking. We had a class for, I think, 10 weeks or something, once a week.’*

However, several obstacles prevented some patients from considering the ITQL, including being unwilling to quit smoking and unconvinced about its benefits. Two participants expressed their thoughts below:

*‘I guess because I know I have to and that I want to.*

*I just haven’t taken that final step.’*

*‘I could really quit on [my] own.’*

Additionally, many participants explained their attitudes through previous experiences that did not work. One participant described:

*‘No, Because I was hearing that for a long time, and that would be the same thing as doing meetings and stuff. And that stuff didn’t work for me – the meetings and talking to counselors. And it doesn’t work for me’.*

### Perceptions about the ITQL among non-users

Some participants perceived that the ITQL would benefit them and were willing to use it to quit smoking. Those willing to try it said ‘it wouldn’t hurt to try’ and they would try it ‘out of curiosity’, hoping to discover something new about themselves.

Others mentioned that regular meetings with their healthcare providers were one of the reasons they were willing to try to quit. Because their providers continually encouraged them to quit smoking. Speaking with a provider directly made them feel cared for, as one participant expressed:

*‘I appreciate the fact that they’re concerned about whether or not I continue with my attempts to stop smoking.’*

Another reason was their determination to try anything it takes to stop smoking:

*‘I might try that, yes. Because right now I’m interested in finding out what I can do to stop smoking’.*

Nevertheless, several participants were not ready to quit but noted that they would be interested in the ITQL when ready:

*‘Yeah, it sounds like something I would be interested in when I’m ready to quit.’*

*‘I will try it. But right now, again, my answer is not right now.’*

*‘If I was to get ready to stop smoking. Yeah.’*

Although some participants were not ready to quit, they expressed a willingness to use the ITQL in the future. Further, after using the program, they reported being willing to recommend the program to others if ‘it had helped them stop smoking’.

### Desired characteristics of the Quitline experience

When queried what would make the Quitline more appealing, participants cited various aspects of their own experiences.

*Peer insights*

Receiving advice from peers and/or former smokers as counselors would increase their trust, as they shared the same experience of smoking. As one participant stated:

*‘Well, if they hadn’t smoked, I wouldn’t be as receptive as I probably would have been.*

*‘Well, as I was saying, it’s a habit. And if you haven’t experienced the habit, there’s book knowledge as opposed to actual knowledge.’*

*Personalization*

The personalization of the smoking cessation plan was believed to enhance patient comfort:

*‘How you talk to someone, they ask you about your tobacco use, then they set up a specific plan for you. I think that would draw a lot of people in.’*

*Empathetic approach*

Expressing empathy toward smokers and demonstrating a willingness to support them can significantly enhance their trust in the initiative. One participant stated:

*‘ … knowing that you got somebody willing to help you, and concern, and show you – and give you help on trying to quit smoking. That’s not easy.’*

According to patients, healthcare providers should avoid applying pressure or force when recommending the program. Feeling ‘rushed’ or ‘pushed’ to adhere to it could lead individuals to regress to a state where they were not ready to quit in the first place. One participant suggested that providers should employ appropriate language and expressions to encourage smoking cessation, such as:

*‘We’re not here to force you to stop smoking, but it’s good for your health, so we’re here to help you.’*

*Outreach approaches*

To effectively promote Quitline services, it is best to use multiple channels. A good advertisement could prove beneficial. Also, using methods such as ‘fliers’, ‘billboards’, or social media, such as ‘TikTok’, can capture the attention of a wider audience:

*‘Fliers or people going into the community talking to people.’*

*‘About now, I feel as though they put something on TikTok … If they happen to have something on there that catches a person’s eye, they would be catching a lot of people’s eyes.’*

*Patient-centered communication*

Healthcare professionals should opt for patient-centered communication strategies to make it easier to connect with the Quitline. Patient-centered communication can be achieved by minimizing ‘too many questions’ and shifting the focus toward two-sided ‘quality, in-depth conversations’ and communicating more ‘frequently’ and ‘constantly’:

*‘I would say for people who are ready to quit like myself, not just bypass the smoking questionnaire. Actually, give out information like ‘How many do you smoke? Or do you know about this program or this and this and that or different options?’ I would say get more in depth with that conversation. Not to drag it on. But you know what I’m saying really [help connect people even to the Quitline person].’*

*‘Constant communication, instead of the three months, I think it should be like once a month instead of – yeah.’*

### Increasing awareness and utilization of the ITQL via the electronic health record

To improve the patient’s connection with the ITQL, using MyChart to promote quit-smoking services was perceived as an excellent way to send information and increase awareness and utilization of the ITQL. Several benefits of using the MyChart portal were reported.

*Collaboration, support and guidance.*

Patients said MyChart messages would facilitate a perception of being under supervision and receiving advice yet still retaining control over quitting. As expressed by one participant:

*‘I think with the MyChart, even though I would wanna do it myself, but setting it up through the MyChart, it makes me personally think like okay, the doctor looked at this, the doctor looked at that, and he thinks with the tobacco specialist, whatever, that this is best. So, that’s something that I would probably follow more.’*

*Convenience and flexibility.*

Many participants expressed their interest in MyChart due to its perceived convenience as an option. They felt that it provided them with a readily accessible resource for moments when they felt ‘ready to quit or [they] noticed something really wrong that [they] need help with, [they] would push on the portal and call people and would have the information in hand’. It also gives the option to access ‘without get[ing] up and do[ing] much or go[ing] and seek[ing] much’. Another participant added:

*‘I think that would be a good idea because if they feel like they want the help, they can always go over to their MyChart and click on that button and say, hey, it’s that time. I think I want help.’*

*Educating and monitoring quitting smoking.*

Patients said utilizing MyChart to promote smoking cessation can enhance awareness as ‘doctors got something to pass on to the people that are not aware of it’. Additionally, it can function as a consistent reminder through ongoing ‘notifications’, aiding individuals in recalling the significance of participating in quitting smoking.

*Facilitating access to care*

Another benefit highlighted by the participants is MyChart’s ability to offer continuous and easily accessible services. As one participant said:

*‘If I called and then somebody didn’t answer, I could just go to MyChart and try that way.’*

## DISCUSSION

This study used surveys and semi-structured interviews to explore: 1) adult smoking patients’ knowledge, attitudes, beliefs, and barriers related to the ITQL; 2) their perception of how to facilitate access and engagement with the ITQL; and 3) the linkage to ITQL via the MyChart electronic health portal. Consistent with research on low-income patients treated in FQHCs, we found high rates of smoking prevalence in the study sample and low rates of ITQL use. Participants indicated individual-level barriers to smoking cessation services and strategies to improve engagement.

Individual-level barriers to ITQL usage centered on participants’ lack of readiness to stop smoking, doubts about its benefits, and prior unsuccessful attempts to quit. These findings are consistent with a systematic review by Pacek et al.^27^, indicating that perceived lack of need and the belief that aid does not help with cessation, are the two most cited reasons for non-engagement with quit-smoking initiatives. In both prior research and this study, a lack of readiness to quit was an important barrier to engaging with the quitline^28^. Additional efforts are needed to increase readiness to quit and the availability of evidence-based and free resources such as the Quitlines^29^.

The study elucidated rich perspectives on enhancing the Quitline program’s appeal and reach. Participants noted the importance of using personalized cessation interventions considering a person’s life circumstances. This is consistent with research showing that tailoring smoking cessation messages or programs to the individuals’ characteristics and preferences are more likely to grab their attention and influence their decision-making^30^. Findings underscored the value of peers who have struggled with smoking and quit as advisors in smoking cessation efforts, aligning with studies on behavior change interventions. For instance, some participants highlighted the importance of being advised by peers and former smokers to increase relatability and trust, consistent with social cognitive theory (SCT). In SCT, individuals are more likely to adopt new behaviors when they see peers successfully engaging in them^31^.

Further, participants noted that empathy, patient-centered communication, and appreciation of the individual’s prior quit attempts can help patients feel motivated to persevere despite previous setbacks. This is consistent with Klemperer et al.^32^ who showed that building an alliance-based empathy with smokers through telephone counseling for smoking cessation is associated with their adherence to the intervention by creating a safe environment for behavior change. Patient-centered communication and empathy approaches align with the principles of motivational interviewing, which have been shown to change behavior, especially in tobacco addiction treatment^33^. Lastly, the suggestion to use diverse outreach tools like pamphlets, billboards, and social media platforms like TikTok is consistent with health communication research, which promotes using several channels and visually engaging platforms for broader reach^34^.

Findings provide initial insight into the role of electronic health records, and MyChart specifically, as a platform to promote smoking cessation services. Participants were favorable to leveraging technology for health promotion. This echoes a growing emphasis on incorporating health information technology to improve engagement, accessibility, and outcomes in healthcare, especially for smoking cessation programs^35^. Participants underscored several advantages, including collaboration, support, convenience, flexibility, education, monitoring, and enhanced access to care. Patients appreciate being supervised and advised (cooperation and support) while retaining agency over the process (convenience and flexibility). This resonates with the principles of patient empowerment and shared decision-making in healthcare^36^. Additionally, the perceptions of MyChart’s ability to provide patient monitoring and education align with the value of continuous engagement and feedback in behavior change management. Furthermore, patients recognized the role of MyChart as a convenient platform to seek support and guidance, highlighting the potential of e-health in facilitating access to smoking cessation programs. Based on these findings, healthcare providers should rely more heavily on MyChart to leverage smoking cessation initiatives and make them available to a broader range of individuals, providing tailored support and guidance, and reinforcing behavior change efforts by promoting patients’ continuous engagement.

### Limitations

The study’s limitations should be considered. First, the relatively small sample size of participants and recruitment methods limit the generalizability of the findings beyond the study’s specific population and context. Second, the findings from the qualitative analyses may be biased by the researchers’ backgrounds and experiences, influencing how they are interpreted and categorized. The patterns and relationships identified in the qualitative data do not reflect causality. Moreover, participants’ reactions may be shaped by their perceptions of what is socially desirable or acceptable, leading to response bias. Furthermore, self-report data in surveys and interviews may be subject to recall biases or question misinterpretation.

## CONCLUSIONS

Tobacco Quitlines are demonstrated effective smoking cessation programs, but they are underutilized. This study revealed the potential positive impact of the electronic health record as a strategy to improve awareness and strengthen utilization of the ITQL by integrating health promotion messages that facilitate individuals’ access to smoking cessation programs. Healthcare providers prioritizing patient smoking cessation and ensuring personalized programs around patient readiness and motivation to quit before introducing them to ITQL and similar interventions, may increase the likelihood of successful quit efforts. Furthermore, several characteristics would render the ITQL more appealing to patients, such as adopting patient-centered communication skills and employing different dissemination approaches about the Quitline. By incorporating these findings into the development and implementation of clinical settings, healthcare providers may improve the effectiveness and outreach of smoking cessation programs.

## Supplementary Material



## Data Availability

The data used to support the findings of this study are included in the article. Raw data and supplementary documents are not publicly available. To request access those data, permission should be obtained from the corresponding author.
