# Supplementary material for: An exploration of attitudes regarding the use of a state tobacco Quitline for smoking cessation among low-income adults with a history of smoking
Source: Tob Prev Cessat. 2024 Nov 5;10:10.18332/tpc/193572. doi: 10.18332/tpc/193572 (PMC11537038; doi:10.18332/tpc/193572)
Supplement: Supplementary file 1 [file TPC-10-50-s1.pdf]

Table 1

Demographic Characteristics of Study Participants (N = 25)

**Characteristic**	**n (%)**
Age (years)	Mean = 52.56 (SD = 10.15)
Race/ethnicity	
Black	19 (76.0)
White	3 (12.0)
Hispanic/Latino	3 (12.0)
Gender	
Male	15 (60.0)
Female	10 (40.0)
Sexual identity	
Lesbian	1 (4.0)
Gay	1 (4.0)
Straight	23 (92.0)
Education	
Less than high school	8 (32.0)
High school	8 (32.0)
Some college	8 (32.0)
Bachelor’s degree or higher	1 (4.0)
Employment	
Employed	9 (36.0)
Unemployed	15 (60.0)
Prefer not to answer	1 (4.0)
Health insurance	
Yes	25 (100.0)
No	0 (0.0)

Table 2

A Summary of Smoking Behaviors and Smoking Health-Related Conditions of Study Participants (N = 25)

Questions	n (%)
1. Have you smoked at least 100 cigarettes in your entire life?	
Yes	25 (100.0)
No	0 (0.0)
2. How many days of the week do you smoke cigarettes?	
6-7 days	22 (88.0)
4-5 days	2 (8.0)
2-3 days	1 (4.0)
1 day	0 (0.0)
3. On average, how many cigarettes do you smoke on the days you smoke?	Mean = 11.32 (SD = 8.06)
4. How soon after waking up in the morning do you have your first cigarette?	
Within 5 minutes	7 (28.0)
Within 5-30 minutes	10 (40.0)
Within 31-60 minutes	4 (16.0)
More than 60 minutes after waking up	4 (16.0)
5. What type of cigarettes do you usually smoke?	
Menthol	21 (84.0)
Regular	2 (8.0)
Both	2 (8.0)
6. How old were you when you first started smoking?	Mean = 16.16 (SD = 3.27)
7. Besides cigarettes, do you smoke any of the following?	
None	20 (83.3)
E-cigarettes	2 (8.3)
Cigars	1 (4.2)
Cigarillos	1 (4.2)
8. If you are in a relationship, does your partner smoke?	
Yes	16 (64.0)

Questions	n (%)
No	5 (20.0)
I am not in a relationship	4 (16.0)
9. How many of your close friends smoke?	
None	1 (4.0)
A few	12 (48.0)
Most	10 (40.0)
All	1 (8.0)
10. Would you say that, in general, your health is?	
Excellent	0 (0.0)
Very good	3 (12.0)
Good	12 (48.0)
Fair	8 (32.0)
Poor	2 (8.0)
11. Have you ever been told by a healthcare provider that you have a smoking-related illness? For example, lung cancer or emphysema.	
Yes	3 (12.0)
No	22 (88.0)
12. Do you have a health condition you have been told is worsened by smoking? For example, diabetes, HIV infection, high blood pressure, lung or respiratory illness, or heart disease.	
Yes	13 (52.0)
No	12 (48.0)

Table 3

A Description of Participants' Smoking Cessation Behaviors, Readiness to Quit, and Use of the Illinois Tobacco Quitline (N = 25)

**Questions**	**n (%)**
1. In the past 12 months, did a doctor, nurse, or other healthcare team member tell you to stop smoking?	
Yes	22 (88.0)
No	3 (12.0)
2. If yes, did they give you information about stop-smoking counseling or medications? *(n* = 22)	
Yes	17 (77.3)
No	5 (22.7)
N/A	3 (12.0)
3. In the past 12 months, have you tried to quit smoking?	
Yes, I have tried to quit	16 (64.0)
No, I have not tried to quit	8 (32.0)
No, I have not tried to quit, but I did cut back or try to cut back	1 (4.0)
4. In the past 12 months, did you use any of the following to help you try to quit? (Check all that apply) (*n* = 16)	
Stop smoking class or support group	2 (12.5)
The Illinois Tobacco Quitline or similar telephone helpline	2 (12.5)
The nicotine patch or gum	8 (50.0)
A stop-smoking medication (Chantix, varenicline, Wellbutrin, or Zyban)	5 (31.3)
Self-help books or pamphlets	3 (18.8)
"Cold turkey"	9 (56.3)
A telephone or internet app	3 (18.8)
Other (praying, chewing tobacco, reading about the harmful effects of smoking as a person with diabetes)	3 (18.8)
None	1 (6.3)
N/A	9 (36.0)

Questions	n (%)
5. If you did not use any stop-smoking medications, why not? (Check all that apply) (*n* = 11)	
My doctor or nurse did not suggest I take them	7 (63.6)
Too many side effects	1 (9.1)
I am worried about becoming addicted to them	1 (9.1)
They are too expensive	1 (9.1)
They are not sold in stores near my home	2 (18.2)
Other (not ready to quit, already on several other medications; did not want to add yet another medication)	2 (18.2)
N/A	14 (56.0)
6. I am ready to quit smoking.	
Completely disagree	1 (4.0)
Disagree	3 (12.0)
Neither agree nor disagree	5 (20.0)
Agree	10 (40.0)
Completely agree	6 (24.0)
7. I feel confident I can quit smoking when I am ready to stop.	
Completely disagree	0 (0.0)
Disagree	9 (36.0)
Neither agree nor disagree	3 (12.0)
Agree	9 (36.0)
Completely agree	4 (16.0)
8. I feel motivated to quit smoking.	
Completely disagree	0 (0.0)
Disagree	5 (20.0)
Neither agree nor disagree	5 (20.0)
Agree	12 (48.0)
Completely agree	3 (12.0)

**Questions**	**n (%)**
9. Have you ever heard of the Illinois Tobacco Quitline?	
Yes	16 (64.0)
No	8 (32.0)
10. Have you ever used the Illinois Tobacco Quitline? (*n* = 16)	
Yes	2 (12.5)
No	14 (87.5)
N/A	9 (36.0)

Note. N/A = not applicable

© 2024 Matthews A.K. et al.
